# Chryseobacterium gleum Causing Healthcare-Associated Pneumonia in an Adult Male With Diffuse Large B Cell Lymphoma

**DOI:** 10.7759/cureus.19297

**Published:** 2021-11-05

**Authors:** FNU Amisha, Tyler Fugere, Jose Caceres, Juan Carlos Rico Crescencio, Nathan Falls

**Affiliations:** 1 Internal Medicine, University of Arkansas for Medical Sciences, Little Rock, USA; 2 Internal Medicine, University of Arkansas for Medical Sciences, Little rock, USA; 3 Pulmonary and Critical Care Medicine, University of Arkansas for Medical Sciences, Little Rock, USA; 4 Infectious Disease, University of Arkansas for Medical Sciences, Little Rock, USA

**Keywords:** diffuse large b cell lymphoma (dlbcl), antibiotic resistant, hospital acquired infection, chryseobacterium gleum, hospital acquired pneumonia, infection control

## Abstract

Chryseobacterium species are recognized as an emerging opportunistic bacterial pathogen in nosocomial settings especially in debilitated or immunosuppressed patients and neonates. The ubiquitous distribution in nature, ability to form biofilms with inherent resistance to broad-spectrum antimicrobials, and lack of clinical studies pose a further diagnostic and therapeutic challenge. This case report describes an elderly male with relapsed diffuse large B-cell lymphoma (DLBCL) status post-chemotherapy and radiation who acquired healthcare-associated pneumonia with sputum isolates showing *Chryseobacterium gleum* and *Stenotrophomonas maltophila. *It also includes a review of literature compiling all the previously reported cases with antibiotic susceptibilities, clinical picture, and treatment outcomes.

## Introduction

*Chryseobacterium gleum* was initially known as *Flavobacterium gleum*, isolated for the first time from high vaginal swabs in 1984 by Homes et al. and was made a separate genus in 1994 [1,2]. *C. gleum* has been associated with various healthcare-associated infections (HAIs) including septicemia, pneumonia, urinary tract infections, wound infections, peritonitis, and meningitis [3-7]. The Sentry Antimicrobial Surveillance program sponsored by JMI Laboratories was first established in 1997 and is the longest-running surveillance program that monitors the changes in resistance patterns of pathogens worldwide through centralized testing. In the 1997-2001 Sentry dataset, Chryseobacterium was recognized for the first time as a medically relevant bacterial species, constituting 0.27% of non-fermentative Gram-negative bacilli found in samples from 16 countries. The highest prevalence was among the elderly. Among the 50 isolates, the most common pathogenic member of this genus was *C. meningosepticum* (24 isolates - 48%) while *C. gleum* (two isolates - 4%) was the most infrequent [8]. In the 2013-2017 Sentry dataset, *C. gleum* constituted only 13 of 151,572 isolates worldwide. Out of 13, 11 were isolated from the United States and 10 of them were HAIs. HAIs are a major cause of morbidity and mortality in the United States, affecting 2 million patients annually, causing 90,000 deaths and loss of 28-45 billion US dollars [9]. Hence, it is important for clinicians to recognize this bacterium especially in immunocompromised patients with hematological malignancies as the infection is largely preventable with infection-control practices.

## Case presentation

This is a case of a 71-year-old hypertensive and diabetic male with coronary artery disease status post percutaneous coronary intervention, idiopathic interstitial lung disease (ILD), and stage IVB diffuse large B-cell lymphoma (DLBCL) treated with chemotherapy and radiation which lead to worsening of his ILD and the requirement of supplemental oxygen and daily low-dose steroid at home. Three months later, he developed a recurrence of his lymphoma and was started on salvage chemotherapy but his lung function continued to decline. Following three cycles of chemotherapy, he presented to the outpatient oncology clinic with a runny nose and nasal congestion, tested positive for rhinovirus, was breathing appropriately, and hence, was managed conservatively. After ten days, he presented to the emergency department with progressive dyspnea and hypoxia with minimal exertion at home with associated subjective fevers.

**Figure 1:**
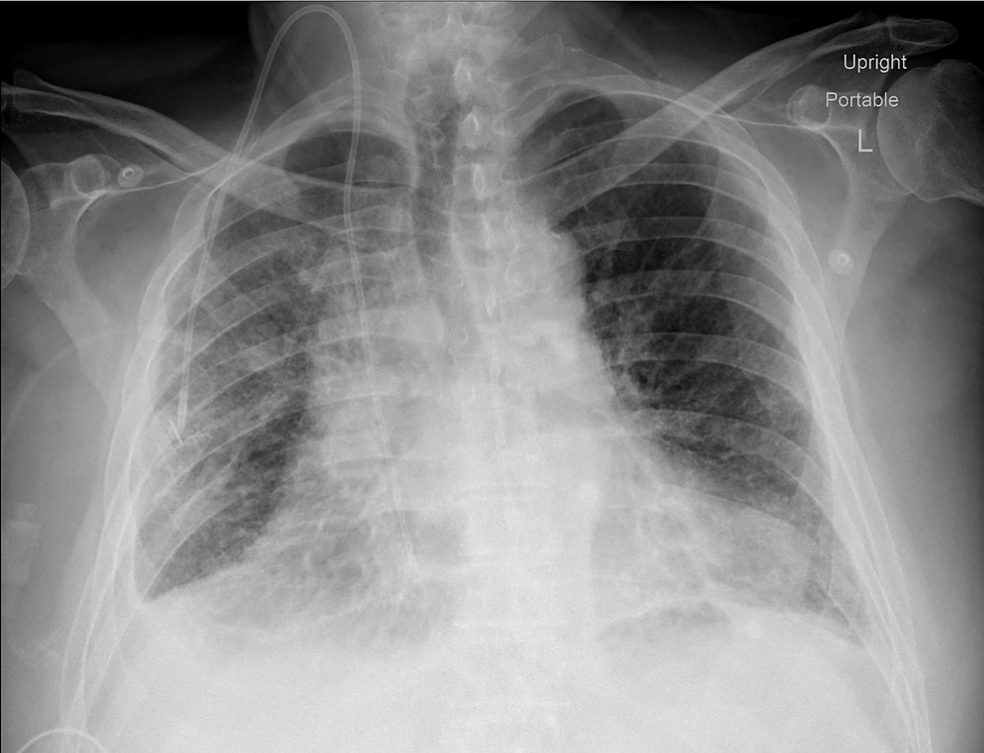
A chest radiograph antero-posterior view - poorly inflated lungs with bibasilar fibro-atelectatic changes and bilateral pleural thickening.

**Figure 2:**
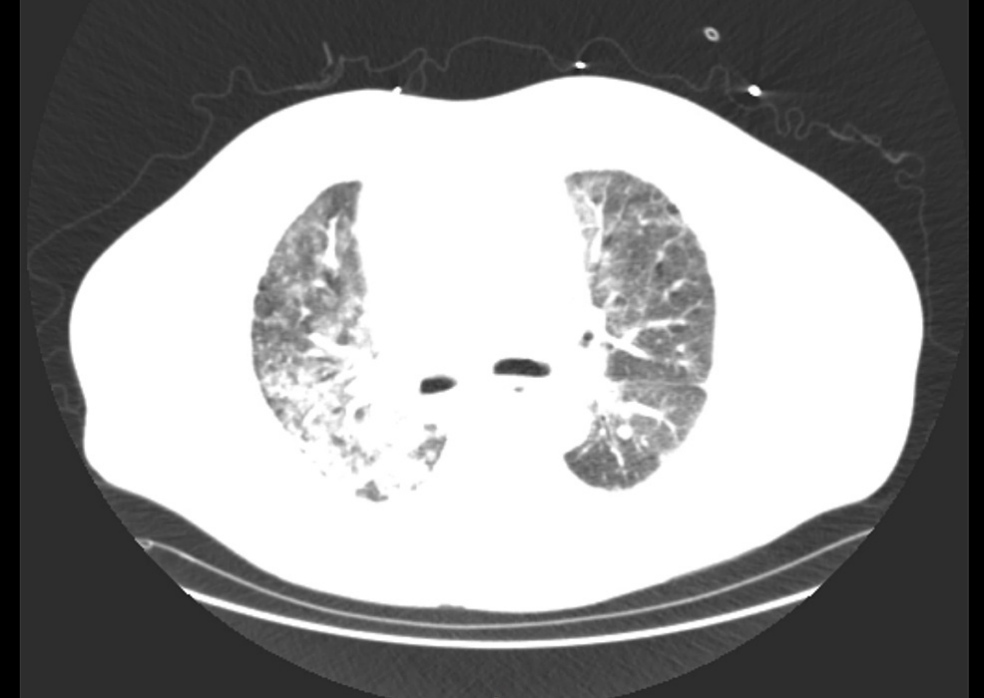
CT chest with contrast-diffuse mosaic attenuation with ground-glass and reticulonodular opacifications on a background of previously visualized fibrotic and bronchiectatic changes with lower lobe predominance and pleural thickening.

On arrival, he was tachypnoeic, tachycardic, febrile, and hypoxic. The examination was significant for rhonchi in bilateral lower lung fields and observed the use of accessory muscles of inspiration. Initial laboratory investigations and chest radiographs are described in Table 1 and Figure 1, respectively. He was admitted to the medical floor and was placed on 4 liters of oxygen via nasal cannula and broad-spectrum antibiotics - azithromycin and cefepime for community-acquired- pneumonia. Computed tomographic imaging of the chest is described in Figure 2.

**Table 1:** Initial laboratory investigations on admission. N/L/M/E/B: neutrophils/lymphocytes/monocytes/eosinophils/basophils; MCV: mean corpuscular volume, MCHC: mean corpuscular hemoglobin concentration; MCH: mean corpuscular hemoglobin; Na/K/Cl/CO_2_: sodium/potassium/chloride/bicarbonate; BUN/Cr: blood urea nitrogen/creatinine; AST/ALT: aspartate aminotransferase/alanine aminotransferase; LD: lactate dehydrogenase; Alk Phos: alkaline phosphatase; RPP: respiratory pathogen panel; BNP: brain natriuretic peptide.

Investigation	Value	Reference range
Hemogram		
Hemoglobin (g/dl)	10.3	13-17
WBC K/µL (N/L/M/E/B %)	6.99 (69.1/18.7/9.4/0.9/0.3)	3.6-9.5 (35-65/23-50/4.6-12/0.5-6.5/0.1-1.1)
Platelets (K/Ul)	180	150-450
MCV (fl)	90.4	80-100
MCH (pg)	27.5	26-33
MCHC (g/dL)	30.5	32-36
Renal chemistry		
Na/K/Cl/CO_2_ (mmol/L)	136/3.6/102/25	135-145/3.5-5.1/98-107/22-32
BUN/Cr (mg/dL)	7/0.4	6-20/0.6-1.3
Liver function test		
AST/ALT (IU/L)	23/24	15-41/4-45
GGT (IU/L)	20	7-50
LD (IU/L)	191	100-248
Alk Phos (IU/L)	59	32-91
Bilirubin, total (mg/dL)	0.9	0.2-1.2
Miscellaneous		
RPP	Rhinovirus positive (as was 10 days ago in-office visit)	
BNP (pg/ml)	41	≤100
D-dimer	936	<250 ng/mL
Procalcitonin (ng/ml)	0.05	0.00-0.10

On the third day of admission, the patient was desaturated to the low 80s while on a non-rebreather mask, requiring him to be placed on a high-flow nasal cannula (HFNC), and he was transferred to the intensive care unit (ICU). Extensive infectious workup was negative: cultures - blood, urine, and fungal; beta-D-glucan, *Aspergillus galactomannan*; cryptococcal, legionella, Histoplasma and Blastomyces antigens; HHV-6, and CMV. His acute decompensation was thought to be from viral pneumonitis leading to ILD flare and he was started on high-dose steroids with notable improvement in his oxygen saturation. Steroids were gradually tapered, and he was transferred out from the ICU on day 16.

**Figure 3:**
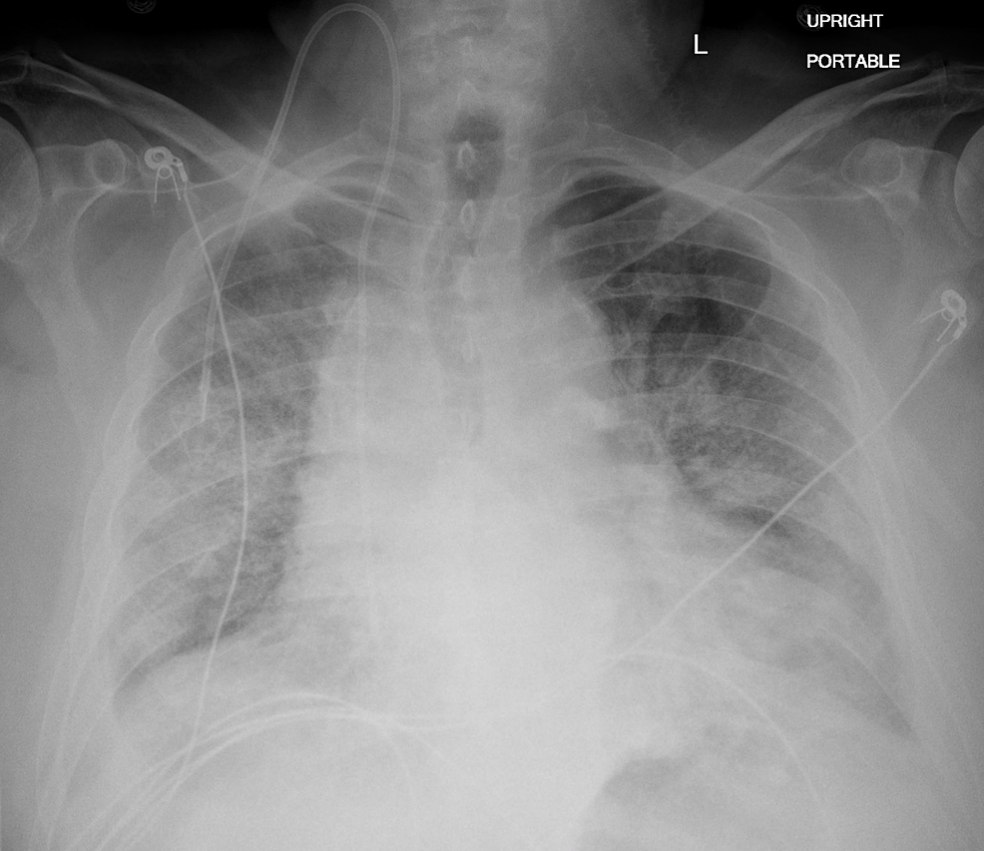
A chest radiograph antero-posterior view - worsening bilateral infiltrates indication acute infectious process.

After five days of being on the floor, his respiratory status declined again. A chest radiograph is described in Figure 3. He was readmitted to ICU, placed on bi-level positive airway pressure (BiPAP), restarted on high-dose steroids and broad-spectrum antimicrobials - cefepime and vancomycin for hospital-acquired pneumonia. Repeat infectious workup was done and respiratory cultures returned positive for *Stenotrophomonas maltophila* and *C. gleum*. He was started on tigecycline and trimethoprim-sulfamethoxazole (SXT) which was changed to levofloxacin after three days because of hyponatremia and once the susceptibility patterns were available (Table 2). His respiratory status continued to worsen, he declined intubation and opted for comfort care measures. He passed away eventually after the removal of BiPAP.

**Table 2:** Summarizing the susceptibility patterns of Chryseobacterium gleum in our study.

Antibiotic	Minimum inhibitory concentration (µg/ml)	Interpretation
Tigecycline	2	R
Amikacin	>32	S
Cefepime	8	S
Ceftazidime	<1	S
Ceftriaxone	8	S
Gentamicin	>8	R
Levofloxacin	0.5	S
Meropenem	<1	S
Piperacillin + tazobactam	<4	S
Tobramycin	>8	R
Trimethoprim + sulfamethoxazole	<2/38	S

## Discussion

Chryseobacterium species are aerobic, catalase-positive, oxidase-positive, non-motile, non-fermentative Gram-negative bacilli (NFGNB) primarily found in soil and water. Environmental studies have shown that they can thrive in chlorinated water and wet surfaces and are not a part of normal flora. Risk factors that are associated with infection include prolonged hospital stay especially in ICUs, indwelling devices, and exposure to broad-spectrum antibiotics. A study from Taiwan has shown that they can form biofilms on medical devices (respirators, intubation tubes, syringes, etc.) or surgically implanted devices (intravenous catheters or prosthetic valves) which contributes to their pathogenic potential [10]. Our patient presumably acquired the infection while in the ICU as his initial respiratory cultures were negative and he responded to steroids, but during the second admission to the ICU, he was unresponsive to steroids and had rapid respiratory deterioration. There were no cultures obtained from the medical devices, but the patient was on contact and droplet precautions during the entire hospital stay which prevented the nosocomial spread of bacteria to other patients in the ICU.

Chryseobacterium spp has been reported to be resistant to several antibiotics such as aminoglycosides, chloramphenicol, colistin, tetracycline, clindamycin, erythromycin, teicoplanin, and beta-lactams. Bellasis et al. found that these bacteria are resistant to beta-lactams as they can chromosomally encode class A beta-lactamases (CGA-1) [11]. In our case, the bacteria were susceptible to most groups of antibiotics including cephalosporins as opposed to the other two reported cases from Unites States [12,13]. Historically, the choice of antibiotics is not well established because of the insufficient data on minimum inhibitory concentration and no Clinical and Laboratory Standards Institute (CSLI) or EUCAST (European Committee on Antimicrobial Susceptibility Testing) guidelines.

In SENTRY studies and most of the reported cases, TMP-SMX and quinolones (Levofloxacin, Gatifloxacin, Garenoxacin) were used to treat the infection. We initially treated our patient with tigecycline and SXT and then Levofloxacin for three days before he opted for comfort care. We did not have a good clinical response because, in most other studies, patients were treated with Levofloxacin for at least 7-10 days. Other causes could be worsening of the underlying ILD due to bacterial proliferation leading to further inflammation and lung damage or poor immune response due to underlying hematologic malignancy. Other studies with documented Chryseobacterium infection have been summarized in Table 3.

**Table 3:** Summarizing and comparing different studies done on Chryseobacterium gleum. AMK: amikacin, AMX: amoxicillin, AMC: amoxicillin-clavulanate, ATM: aztreonam, CAZ: Ceftazidime, CFP: cefoperazone, CFZ: cefazolin, CIP: ciprofloxacin, CLI: clindamycin, CRO: ceftriaxone, CST: colistin, CTX: Cefotaxime, DAP: daptomycin, DOX: doxycycline, DOR: doripenem, ERY: erythromycin, FEP: cefepime, FOX: cefoxitin, GEN: gentamicin, IPM: imipenem, LVX: levofloxacin, MIN: minocycline, MEM: meropenem, PIP: piperacillin, SAM: ampicillin-sulbactam, SXT: trimethoprim-sulfamethoxazole, TGC: tigecycline, TOB: tobramycin, TET: tetracycline, TZP: piperacillin-tazobactam, TIC: ticarcillin, TIM: ticarcillin-clavulanic acid, VAN: vancomycin, NIT: nitrofurantoin.

Study	Type of study	Date	Region	Sex/age	Disease/site of source	Comorbidities	Susceptibilities	Treatment	Duration	Response
Nemli et al. [3]	Case report	2015	Croatia	Female/35	Pneumonia/blood and tracheal aspirate	Hepatic lesion Malnutrition	R: CST, DAP, IPM, MEM, VAN S: CAZ, CIP, FEP, TGC, TZP	TZP	12 days	Positive
Jain et al. [4]	Case report	2017	India	Male/62	Sepsis and pneumonia Blood and tracheal aspirate	CKD CAD s/p CABG MVA with tentorial bleed	R: AMX, CAZ, CFP, CLI, CRO, CST, CTX, DOX, ERY, FEP, GEN, IPM, MEM, TOB S: AMK, CIP, DOX, LVX, MIN, SXT. TZP, VAN	LVX	10 days	Negative
Rajendran et al. [5]	Case report	2016	India	Male/58	Metabolic encephalopathy/urine	Diabetic CKD CVA	R: GEN, AMK, MEM, ATM, CST, S: IPM, MIN, LVX, CIP, SXT	CIP	6 days	Lost to follow up
Garg et al. [6]	Case report	2013	India	Male/48	Pyonephrosis/urine and percutaneous nephrostomy fluid	B/l renal and ureteric calculi	R: AMX, CTX, CAZ, CFP, FEP, IPM, MEM, GEN, TOB, AMK, CIP, ERY S: TET, MIN, NIT, TZP	TET	7 days	Positive
Lo and Chang [10]	Cases series- 14 isolated	2014	Taiwan	NA	The most common was urine (35.7%, 5/14) followed by sputum (28.6%, 4/14)	NA	R: AMK, AMS, CAZ, CFZ, CRO, CST, FEP, FOX, GEN, IPM, PIP, TZP S: CIP, MIN, SXT, TGC	NA	NA	NA
Anson et al. [12]	Case report	2020	United States	Female/76	CLABSI/blood	CKD stage3 CHF vascular dementia C. diff Megacolon s/p total colectomy and ileostomy	R: SAM, ATM, CFZ, CRO, GEN, MEM, TZP S: LVX, SXT	Lvx	14 days	Positive
Tsouvalas et al. [13]	Case report	2019	United States	Male/61	Pneumonia/sputum	Supraglottic laryngeal carcinoma s/p chemoradiation in remission COPD	R: AMK, ATM, CAZ, CRO, FEP, GEN, IPM, MEM, TOB S: SXT, TZP	SXT	7 days	Positive
Abdalhamid et al. [14]	Case report	2016	Saudi Arabia	Newborn	Pneumonia/endotracheal aspirate	Nephrotic syndrome	R: AMK, CAZ, CIP, CST, FEP, GEN, IPM, MEM, TGC, TZP, VAN S: LVX, MIN, SXT	LVX	16 days	Positive
Arouna et al. [15]	Case report	2017	West Africa	Male/68	UTI/urine	After prostatectomy	R: MEM, ATM, CTX, TIC, TIM S: PIP, CAZ, FEP, CIP, IPM	CIP + AMK	NA	Positive
Virok et al. [16]	Case series-3 patients	2014	Hungary	Newborn	Pneumonia/stomach content	NA	R: AMK, DOR, GEN, IPM, MEM, TOB, TZP S: CAZ, CIP, FEP, LVX	CIP	NA	Positive
Ramya et al. [17]	Case report	2015	India	Male/62	Renal calculi/hydronephrosis urine	CKD diabetes hypertension	S: TZP, CTX, CAZ R: NIT, TOB, GEN, AMK	TZP	7 days	Positive
Rawat et al. [18]	Case series	2017	India	Paediatric	NA	Chronic granulomatous disease	R: NA S: MIN, SXT. TZP	TZP + SXT	NA	Positive
Mirza et al. [19]	Case series	2018	Turkey	Paediatric	NA	Most common cystic fibrosis	R: AMK, GEN, IPM, MEM S: CAZ, CIP, FEP, LVX, SXT, TZP	NA	NA	NA
Singal et al. [20]	Case report	2017	India	Male/64	Sepsis/blood	Diabetes hypertensive COPD	R: IPM, CAZ, FEP, CRO, AMC S: AMK, CIP, LVX, TZP, TET, SXT	LVX	7 days	Positive

## Conclusions

*Chryseobacterium gleum* is an emerging opportunistic/nosocomial pathogen in critically ill patients in ICUs, on mechanical ventilation, and receiving broad-spectrum antibiotics. We emphasize that isolation of this rare organism from clinical specimens should prompt an in vitro susceptibility pattern testing to optimize the treatment as soon as possible for better clinical outcomes. Due to limited susceptibility, the chances of *C. gleum* becoming a major infectious threat are high, especially in immunocompromised patients with hematological malignancies. The choice of effective drug therapy for empiric treatment may be difficult due to the scarcity of succinct evidence-based data, but based on the review of literature, levofloxacin or TMP-SMX are feasible empiric options followed by piperacillin-tazobactam. Infection control practices including hand hygiene, cleaning supplies with alcohol-based hand sanitizer, aseptic procedures, and airborne precautions are effective preventative measures to control spread.
